# A Method for Preparing Superhydrophobic Paper with High Stability and Ionic Liquid-Induced Wettability Transition

**DOI:** 10.3390/ma14164638

**Published:** 2021-08-18

**Authors:** Shangjie Jiang, Shisheng Zhou, Bin Du

**Affiliations:** 1 Faculty of Printing, Packaging Engineering and Digital Media Technology, Xi’an University of Technology, Xi’an 710048, China; zhoushisheng@xaut.edu.cn (S.Z.); dubin@xaut.edu.cn (B.D.); 2 Shaanxi Provincial Key Laboratory of Printing and Packaging Engineering, Xi’an University of Technology, Xi’an 710048, China

**Keywords:** superhydrophobic paper, ionic liquid-responsive properties, stability, transparency

## Abstract

In this study, the polymer PTSPM-PMETAC with anion adsorption properties was prepared by a one-step method, then the amino-modified nano-SiO_2_ was grafted onto the polymer to improve the roughness of the surface and enhance the stability of superhydrophobic properties, and a high-stability superhydrophobic paper with ion-induced wettability transition properties was successfully prepared. The study found that the paper can realize the reversible control of surface wettability through the exchange between the anions PF_6_^−^ and Cl^−^ adsorbed on the surface of PMETAC, and further investigation of the effect of different solvents on the ion exchange properties found that water was the poor solvent for ion exchange, while the mixtures of methanol, acetone, and methanol & water were the good solvent. On the whole, the preparation of superhydrophobic paper by this method not only simple in preparation process, low in cost and strong in universality, but also the prepared superhydrophobic paper has high transparency and good stability, which has great application potential in industrial production.

## 1. Introduction

Due to its rich resources, low price, easy recycling and degradation, paper is widely used in various fields of life as a kind of environmentally friendly material [1,2,3]. However, the hydroxyl group contained in the paper fiber makes it exhibit extremely strong hydrophilic properties, which greatly limits its application value. Therefore, how to make paper have superhydrophobic properties through hydrophobic modification has become the key to expanding its application range and improving its application value [4,5,6]. Generally speaking, the wettability of the substrate surface is determined by its surface chemical composition and microscopic rough structure, but external stimulus factors such as light, electricity, temperature, and solution properties can also have an important impact on the wettability. Especially in actual production applications, controlling the wettability of the substrate surface by changing external stimuli to prepare a functional superhydrophobic surface has become a key area of attention in modern industrial production applications [7,8]. The basic principle of realizing the wettability transformation of the substrate surface by adjusting the external stimulus is that the chemical composition, surface structure and surface free energy of the substrate surface active components will change correspondingly under the external stimuli, which will cause the substrate surface reversible transformation of wettability [9,10]. According to the existing research, the preparation methods of functional superhydrophobic paper, such as electric response, pH response, temperature response, light response, often have some problems, such as high requirements for preparation conditions, more complicated preparation processes, and poor transparency and stability of the superhydrophobic surface [11,12].

For example, the related studies have proposed some preparation methods for functional superhydrophobic surfaces, but they all have some limitations. Firstly, by depositing a layer of long-chain molecules with hydrophilic ends on the gold surface, a superhydrophobic surface can be prepared that can realize the reversible change of surface wettability through electrical induction. However, the more expensive raw materials, poor flexibility of the substrate, as well as the difficulty of processing and recycling and degradation limit its application value to a certain extent [13]. Secondly, cellulose stearoyl ester (CSE) solution and nano particles were used to treat the paper to prepare intelligent superhydrophobic paper with temperature-responsive properties. However, due to the lack of adhesion between polymer and nano particles, the polymer or nano spheres were easy to fall off, resulting in the instability of superhydrophobic surface [14]. Thirdly, Poly (diallyl dimethyl ammonium chloride) (PDDA) and poly-4-styrene sulfonate (PSS) were used to self-assemble layer by layer on a silicon substrate to obtain a polyelectrolyte multilayer film, and the surface contact angle can be changed between 19° and 114° by changing the anions adsorbed by the outermost PDDA. However, due to the insufficient surface roughness of the material, the variable range of the surface wettability was very small, and the control of the surface wettability through ion exchange was also relatively limited. In addition, the reaction conditions of this method were relatively harsh and the preparation process was relatively complicated, and it was difficult to apply to the mass production [15]. Fourthly, after the surface of azobenzene polyelectrolyte with photo induced reversible wettability was prepared by electrostatic self-assembly technology, the azobenzene molecules were modified on the rough substrate, and the reversible conversion between superhydrophobicity and superhydrophilicity was realized by visible light and ultraviolet light alternative irradiation [16].

In recent years, the application of ionic liquids in industrial production has had a great impact on people’s daily life, and has also attracted widespread attention in the field of academic research [17,18]. Because of its excellent physical and chemical properties, such as low volatility, low flammability, strong thermal stability and strong surface activity [19,20,21,22,23,24], it is easy to change the solution properties by changing the content and combination of ions in the solution, which makes ionic liquids have a wide range of applications in the fields of reaction solvents, reaction catalysts, biochemistry, electrochemistry, analytical chemistry, pharmacy and medicine [25,26,27,28,29,30]. Inducing the transformation of paper surface wettability through ionic liquids is an important application of ionic liquids in the research field of functional superhydrophobic. It has become a research hotspot due to its advantages of lower equipment requirements, faster response speed, and relatively simple preparation process. In this study, based on the existing preparation methods of functional superhydrophobic surfaces, the first step was used to prepare methacryloyloxyethyl trimethy lammonium chloride (METAC) and 3-(Methacryloxy) propyltrimethoxysilane (TSPM) polymer PTSPM-PMETAC, and then use APTES to aminate silica nanoparticles to improve the surface roughness of the paper and enhance the adhesion of the polymer to SiO_2_. Finally, after the prepared polymer was grafted with amino-modified SiO_2_ and sprayed on the surface of the paper, superhydrophobic paper with ionic liquid-induced wettability transition properties was successfully prepared. It was found that the surface wettability of the paper could be controlled reversibly by changing the anions adsorbed by PMETAC. Using this method to prepare functional superhydrophobic paper, not only the preparation process was relatively simple, the preparation efficiency was high, the universality was strong, but also the prepared paper surface coating had good stability and high transparency, which showed a strong application potential in practical production and application.

## 2. Experimental Section

### 2.1. Experimental Materials

The relevant information of the main experimental materials used in this study was as follows: APTES (Union Silicon), methacryloyloxyethyl trimethylammonium chloride (METAC), 3-trimethoxysilyl propyl methacrylate (TSPM), triethylamine (Tianli), sodium hexafluorophosphate (NaPF_6_) (Xuejia fluorosilicone), nano-SiO_2_ (Aladdin), sodium chloride (NaCl), sodium bisulfite (NaHSO_3_), ethylene glycol monomethyl ether, aluminum oxide (Al_2_O_3_), potassium bromide, azobisisobutyronitrile (AIBN) (Four Hervey), anhydrous ethanol (Miura), sandpaper (Fuji Star). It should be noted that all these chemicals have not been further purified before using.

### 2.2. Experimental Procedure

**Figure 1:**
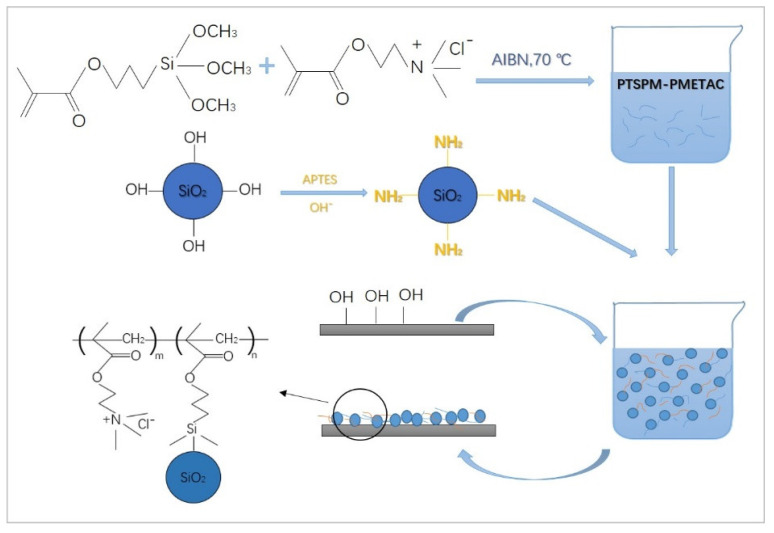
Preparation process of ion-responsive superhydrophobic paper.

The main experimental procedure of preparing superhydrophobic paper in this study is shown in Figure 1, and the main steps are as follows:

#### 2.2.1. Preparation of Polymer Coating PTSPM-PMETAC with Anion Adsorption Properties 

**Figure 2:**
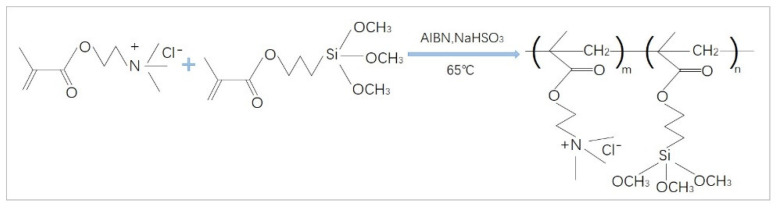
Synthetic route of polymer PMETAC-PTSPM.

First of all, the polymerization inhibitor was removed by filtering the TSPM with an alumina chromatography column. Afterwards, at room temperature, TSPM (0.03 mol, 7.45 g), METAC (0.03 mol, 6.23 g), AIBN (0.002 mol, 0.33 g) and NaHSO_3_ (0.002 mol, 0.21 g) were added to the ethylene glycol methyl ether (100 g) in a flask, and after bubbling nitrogen into the flask for 45 min to reduce the oxygen, seal the flask and freeze the flask, and then purge with nitrogen, repeated this operation three times. Finally, placed the flask in an oil bath heated at 70 °C and magnetically stirred for 5 h to obtain a colorless and transparent polymer coating PTSPM-PMETAC (Figure 2).

#### 2.2.2. Preparation of Amino-Modified Nano-SiO_2_

At first, SiO_2_ (5 g) was added to absolute ethanol (200 mL) for ultrasonic dispersion treatment for 30 min to obtain a silicone ethanol dispersion, and triethylamine was added dropwise to the dispersion to make its pH value reach 9. Secondly, the coupling agent APTES (5 g) was added to the dispersion, and the flask was vacuumized, sealed, frozen and filled with nitrogen. This step was repeated three times. After the flask was heated to 80 °C in water bath and stirred by magnetic force overnight, the amino modified nano-SiO_2_ dispersion was obtained. Afterwards, the obtained nano-SiO_2_ dispersion was centrifuged at 3000 rpm/30 min, after washing and drying the precipitate with absolute ethanol, the purified amino-modified SiO_2_ was obtained. Finally, powdery amino-modified nano-SiO_2_ was obtained after drying the purified the amino-modified SiO_2_ at 80 °C/12 h and grinding it.

#### 2.2.3. Preparation of Superhydrophobic Paper with Ionic Liquid-Responsive Properties 

In the first place, 2 g of amino modified nano-SiO_2_ was added into the polymer PTSPM-PMETAC and stirred by magnetic force for 3 h to obtain the modified solution PMETAC-PTSPM /SiO_2_-NH_2_. Then,10 g of modified solution PMETAC-PTSPM /SiO_2_-NH_2_ was taken and 20 cm × 20 cm (about 2.5 g) of paper was completely immersed in it until the paper fully absorbed the modified solution. Finally, the superhydrophobic paper with ionic liquid-responsive properties can be prepared after taking out the paper and drying it in an oven at 80 °C for 3 h.

### 2.3. Characterization

The paper used in this study was German Duni brand wood pulp paper, and the paper size was 40 cm × 40 cm, the weight of single sheet paper was about 9.5 g, the thickness was 0.48 mm, and the surface flatness was about 87 s. The contact angle measuring instrument DS100 was used to measure the contact angle and rolling angle of the droplet on the surface of the substrate, the droplet used in the experiment was 5 μL, and 10 different points were selected for each sample and the average value was recorded. The JSM-6701F field emission scanning electron microscope (Shanghai Zeiss Optical Instrument International Co., Ltd., Shanghai, China) was used to observe the scanning electron microscope (SEM) image, and the sample was sprayed with gold before observing the micro-morphology of the sample. The Bruker VECTOR-22 infrared spectrometer (Shimadzu Corporation, Kyoto, Japan) was used to test Fourier Infrared Spectroscopy (FT-IR). The (Axis Ultpa) X-ray photoelectron spectrometer was used for the XPS test, Al/Kα (1486.71 eV) (Kratos, Manchester, United Kingdom) was used as the ray, and it was operated under the conditions of a current of 10 mA and a voltage of 10 KV in the experiment. STA449CTG thermogravimetric analyzer (Netzsch Scientific Instruments Trading (Shanghai) Co., Ltd., Shanghai, China) was used to carry out the thermogravimetric test (TG) of the samples, and the experiment was carried out in an air environment, the heating rate was 10 °C/min. Integrating sphere spectrophotometer (American X-Rite Co., Ltd., Granville, MI, America) was used to test the reflection spectrum, and the wavelength range tested in the experiment was 400 nm–700 nm. In the weather resistance test, the prepared modified paper was exposed to outdoor air for 3 months, and 5 different points were randomly selected at different time to test the contact angle of the droplet on the paper surface, and the average of the contact angle was recorded. In the abrasion resistance test, after the bottom end of the 500 g weight was pasted with the sandpaper (the sandpaper model was 2000 mesh), the weight was rubbed back and forth in the same horizontal direction at a speed of 2 cm/s with a friction destruction length cycle of 20 cm. 5 different points were randomly selected to test the contact angle of droplets on the paper surface in the experiment, and the average value of the contact angle was recorded.

## 3. Result and Discussion

### 3.1. Synthesis of Polymers and Modified Coatings

**Figure 3:**
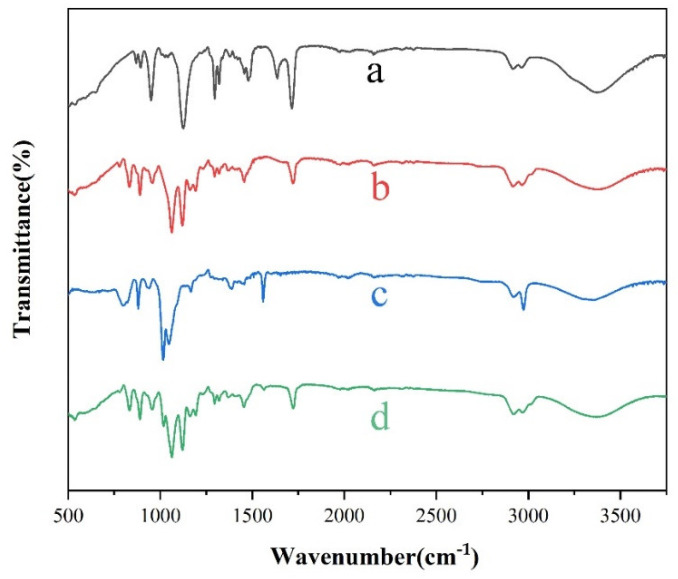
Fourier infrared spectrum of (a) METAC; (b) Ion-responsive polymer PTSPM-PMETAC; (c) Amino modified SiO_2_; (d) Ion-responsive superhydrophobic coating PTSPM-PMETAC/SiO_2_-NH_2_.

Figure 3 shows the infrared spectra of polymers and superhydrophobic coatings. Curve a is the infrared spectrum of METAC. As shown in the Figure 3, the stretching vibration absorption peak of methyl appears at 2975 cm^−1^, the characteristic absorption peak of methylene stretching vibration appears at 2913 cm^−1^, the stretching vibration absorption peak of CN appears at 1119 cm^−1^, the flexural vibration peak of CO appears at 1292 cm^−1^, the flexural vibration absorption peak of C=C appears at 1635 cm^−1^, and the stretching vibration absorption peak of C=O appears at 1716 cm^−1^. Curve b is the infrared spectrum of the polymer PMETAC-PTSPM. In addition to the basic characteristic peaks of curve a, 815 cm^−1^ is the symmetrical stretching vibration peak of Si-C, 1063 cm^−1^ is the stretching vibration peak of Si-O-C, and the characteristic peak of C=C disappears near 1635 cm^−1^. These changes in the infrared spectrum imply the formation of the polymer PTSPM-PTSPM. Curve c is the infrared spectrum of amino-modified SiO_2_. The antisymmetric stretching vibration peak of Si-O-Si appears at 1028 cm^−1^, the characteristic absorption peaks of methyl and methylene stretching vibration appears at 2917 cm^−1^ and 2910 cm^−1^ respectively, and the bending vibration peak of N-H appears at 1551 cm^−1^, the appearance of the above peaks proves the successful modification of SiO_2_. Curve d is the infrared spectrum of PMETAC-PTSPM/SiO_2_-NH_2_. The stretching vibration peak of Si-OC appears at 1062 cm^−1^, the stretching vibration absorption peak of CN appears at 1119 cm^−1^, and the antisymmetric stretching vibration peak of Si-O-Si in SiO_2_ appears at 1024 cm^−1^. As shown in the Figure 3, the characteristic peaks of curves b and c can be observed in the FTIR spectrum of PMETAC-PTSPM/SiO_2_-NH_2_. The results of infrared spectroscopy indicate the synthesis of the polymer and the successful introduction of amino-modified SiO_2_ in the polymer [31,32].

### 3.2. Analysis of Surface Elements of Modified Paper

**Figure 4:**
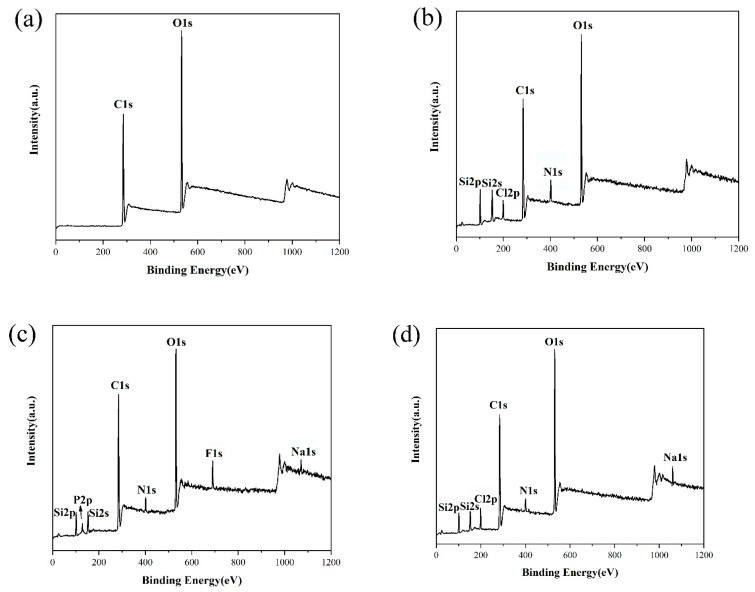
XPS spectra of (**a**) Original paper; (**b**) Modified paper; (**c**) Modified paper soaked in NaPF_6_; (**d**) Modified paper after soaking in NaPF_6_ and NaCl solutions.

Figure 4 shows the results of using XPS to analyze the chemical composition of the paper surface before and after polymer grafting to verify whether PMETAC-PTSPM / SiO_2_-NH_2_ has been successfully grafted to the paper surface. Figure 4a is the XPS spectrum of original paper. From the figure, only the peaks corresponding to C and O at 283 eV and 530 eV can be observed, which are mainly derived from the fibers in the paper. For the modified paper, as shown in Figure 4b, in addition to the C and O elements on the surface, the characteristic peaks of Si2p and Si2s appear at 100 eV and 151 eV respectively, the characteristic peak of Cl2p appears at 198 eV, and the characteristic peak of N1s appears around 401 eV. Among them, N and Cl elements mainly come from the polymer PMETAC, and Si elements mainly come from PTSPM and SiO_2_. The above phenomena can prove the successful modification of the paper [33].

**Figure 5:**
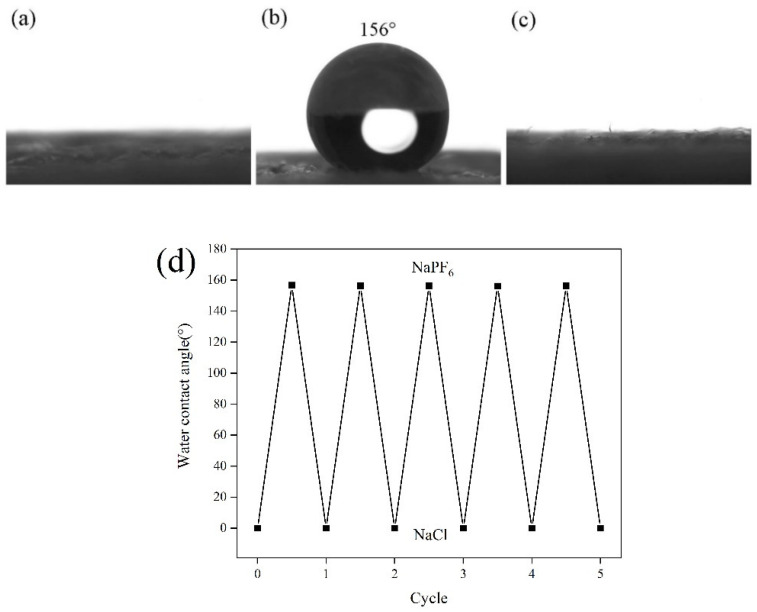
Contact angle of (**a**) Modified paper; (**b**) Modified paper after soaking in NaPF_6_; (**c**) Modified paper after soaking in NaCl solution; (**d**) Modified paper after soaking in NaPF_6_ and NaCl solutions.

Different anions have different surface energy. By changing the anions adsorbed by PMETAC on the paper surface, the paper surface energy and the wettability of the paper surface can be changed. In this study, PF_6_^−^ ion and Cl^−^ ion were selected for ion exchange with the surface of PMETAC to test the influence of different anions on the wettability of the paper surface. Firstly, the hydrophobicity of the modified paper was tested, and it was found that the modified paper was hydrophilic before being treated, as shown in Figure 5a. Afterwards, the modified paper was immersed in a methanol solution of 0.2 mol/L NaPF_6_ for 2 h, and then washed with deionized water and dried for XPS test. The experiment found that in addition to elements C, O, N, and Si on the paper surface, the characteristic peak of Na1s appeared at 1071 eV, the characteristic peak of P2p appeared at 136 eV, and the characteristic peak of F1s appeared at 688 eV (Figure 4c). At this time, due to the adsorption of the low surface energy F element, the paper surface has superhydrophobic properties, and its contact angle is 156°, as shown in Figure 5b. The paper with superhydrophobic properties was further immersed in a NaCl solution of methanol (with a concentration of 0.2 mol/L) for 2 h, and the XPS test was performed after drying. The experiment found that the surface elements of the paper changed to C, O, N, Si, Na, Cl, and the F element with low surface energy was replaced by the Cl element (Figure 4d), which made the paper surface exhibit superhydrophilic properties. The contact angle of the paper surface at this time was shown in Figure 5c. As shown in Figure 5d, it was found that the paper surface can still achieve the transition between superhydrophobic and superhydrophilic after repeating the above soaking process 5 cycles, which showed that the reversible control of the wettability of the paper surface can be achieved by ion exchange between PF_6_^−^ and Cl^−^.

**Figure 6:**
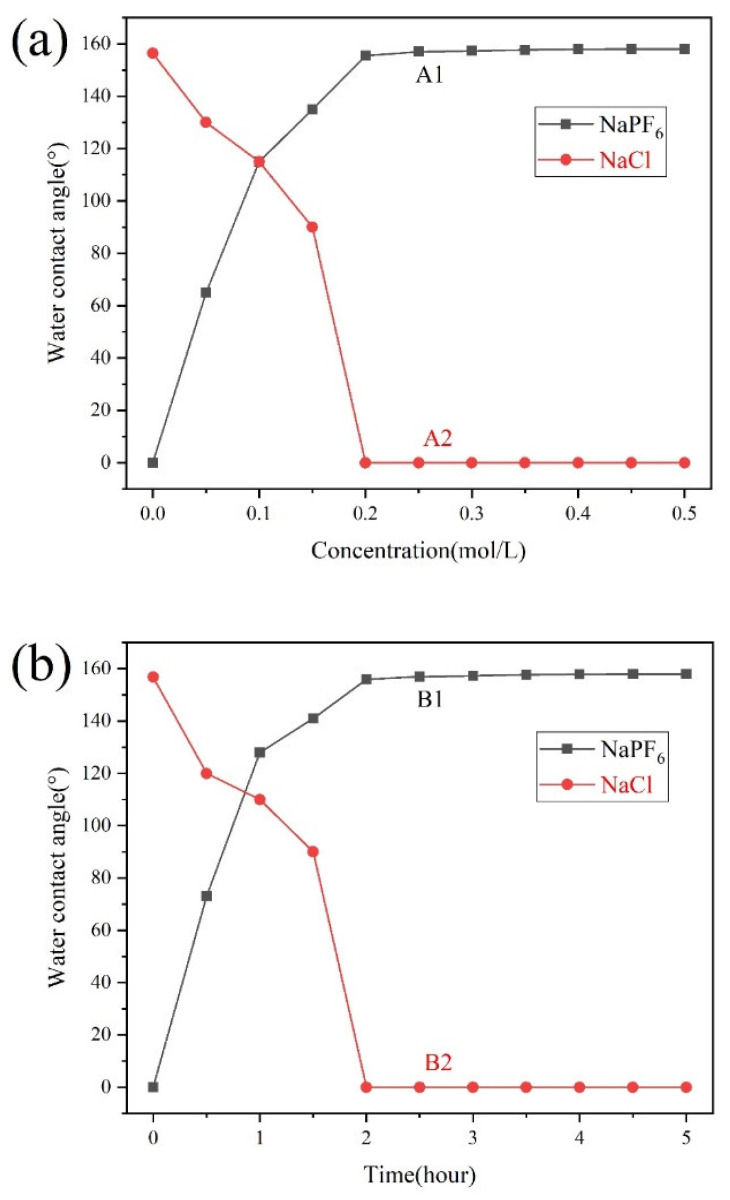
(**a**) The influence of the concentration of anion solution on the contact angle of the paper (soaking time is 2 h); (**b**) the effect of soaking time on the contact angle of the paper surface (the selected anion solution concentration is 0.2 mol/L).

The concentration of the anion solution and the soaking time will have an important impact on the anion adsorption capacity of the polyelectrolyte molecular brush PMETAC, thereby affecting the contact angle of the paper surface [34,35]. The solvent used in this experiment was methanol. Figure 6a shows the effect of the anion solution concentration on the contact angle of the paper surface (soaking time 2 h). It can be seen from the curve A1 when the modified paper was immersed in 0.2 mol/L NaPF_6_ solution, the surface of the paper acquired superhydrophobicity with a contact angle of 156°. And as the solution concentration continued to increase, the contact angle of the paper surface did not change significantly. While the superhydrophobic paper adsorbed PF_6_^−^ anions was immersed in NaCl solutions of different concentrations for ion exchange, it was found when the concentration of the NaCl solution was 0.2 mol/L, the surface of the paper became superhydrophilic (curve A2). Therefore, considering the cost control factors, the best concentration of anion solution was 0.2 mol/L. Figure 6b shows the effect of soaking time on the contact angle of the paper surface (the selected anion solution concentration was 0.2 mol/L). It can be seen from the figure that when the modified paper is soaked in NaPF_6_ solution for 2 h, the surface of the paper has obtained superhydrophobicity (curve B1); and when the superhydrophobic paper adsorbed PF_6_^−^ anions was soaked in NaCl solution for 2 h, the paper became superhydrophilic (curve B2). By further increasing the soaking time, it was found that there was almost no effect on the contact angle of the paper surface, and long-term soaking may also damage the mechanical properties of the paper, so the best soaking time was 2 h. Based on the above analysis, the optimal anion solution concentration was 0.2 mol/L, and the immersion time was 2 h.

Many studies have found that the nature of the solvent had a great impact on the ion exchange on the paper surface [36,37]. Considering that superhydrophobic paper will be inevitably exposed to various solvents in actual use, this will have a certain impact on the surface properties of the paper. In this study, four solvents: water, methanol, acetone, and methanol/water (volume ratio 1:1) mixed solvent, were selected to test their effects on ion exchange on superhydrophobic surface. During the experiment, the concentration of all solutions for ion exchange was adjusted to 0.2 mol/L. In the specific operation, the modified paper was immersed in different ionic solutions, and then cleaned with deionized water after being taken out, and dried at the temperature of 75 °C, and this process was repeated 3 times. In addition, the soaking time of paper in sodium hexafluorophosphate (NaPF_6_) and sodium chloride (NaCl) was also set to 2 h.

**Figure 7:**
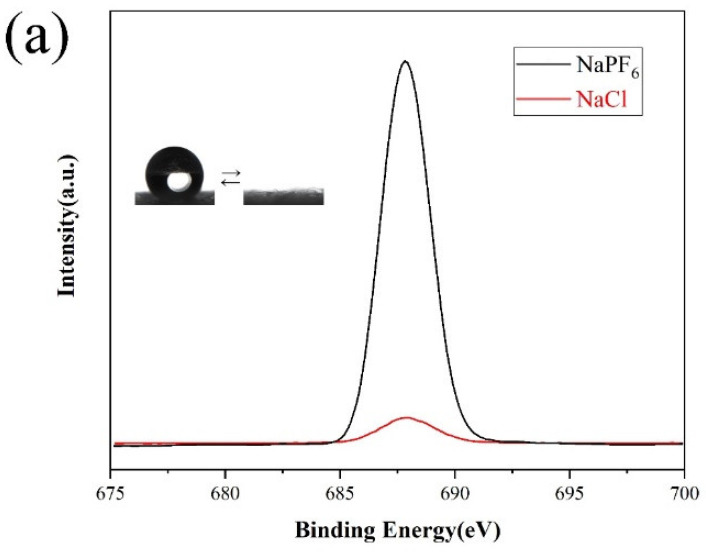

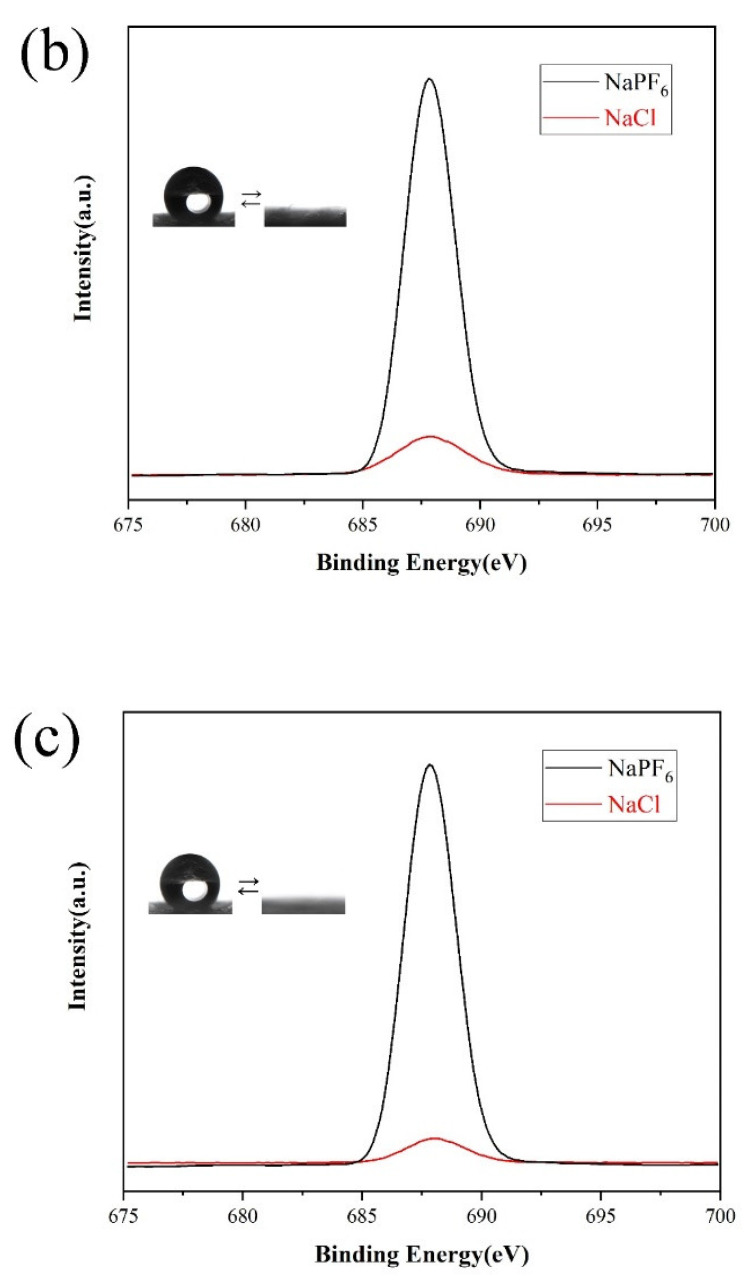

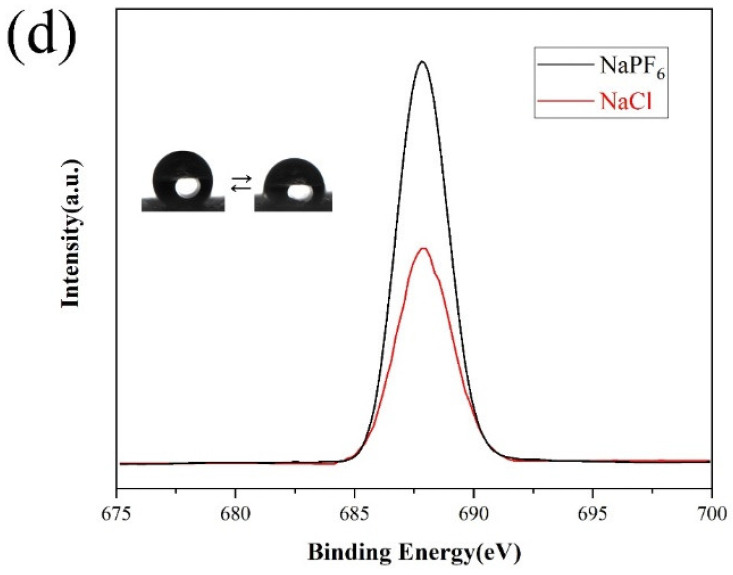
XPS spectra of F element on the surface of anions dispersed on paper treated with different solvents (**a**) Acetone; (**b**) Water/methanol (*v*/*v*: 1/1); (**c**) Methanol; (**d**) Water.

Figure 7 shows the characterization results of the chemical composition on the surface of modified paper grafted with PMETAC before and after ion exchange in different solvents by XPS spectra. It can be seen from Figure 7a–c that when the solvent was methanol and acetone, the ion exchange between PF_6_^−^ and Cl^−^ on the modified paper surface was relatively complete. In this process, the modified paper surface can realize the transition from superhydrophobic to superhydrophilic properties. Similarly, when the solvent was a 1:1 mixture of methanol and water, the PMETAC adsorbed on the surface of the modified paper with PF_6_^−^ was exchanged with Cl^−^, and the concentration of the F element on the surface of the paper was greatly reduced. This reflected that the ion exchange of PF_6_^−^ and Cl^−^ in the mixture of methanol and water was also relatively sufficient, and the wettability of the paper surface can also achieve the transition between superhydrophobicity and hydrophilicity. As shown in Figure 7d, under the condition of using water as the solvent, when the surface of the paper was ion-exchanged with PF_6_^−^, it showed a strong characteristic peak of Fls, which indicated that PF_6_^−^ ions were adsorbed on the surface of the paper; and when the surface of PMETAC adsorbed PF_6_^−^ ion was exchanged with Cl^−^, although the atomic concentration of F element on the surface was reduced, its quantity was still relatively large. These XPS test data showed that the ion exchange from PF_6_^−^ to Cl^−^ was not sufficient, and there was still a large amount of PF_6_^−^ remaining on the surface of the paper. From the perspective of the realization of the superhydrophobic properties of the paper, it is precisely because of the existence of PF_6_^−^ ion, a substance with low surface energy, that the surface of the paper still has strong hydrophobic properties.

In the solvents of different properties, through ion exchange experiments between PMETAC and PF_6_^−^ and Cl^−^, it was found that water was the poor solvent for ion exchange, while the mixture of methanol, acetone, and methanol & water were all the good solvent. The reason for this result, may be that PF_6_^−^ is a hydrophobic ion. When the solvent is water, compared to Cl^−^ ions, PF_6_^−^ ions are more easily absorbed by PMETAC molecules, resulting in incomplete ion exchange from Cl^−^ to PF_6_^−^ in the aqueous solution; And when the solvent is an organic solution such as methanol, acetone, a mixture of methanol and water, PF_6_^−^ ions will be more stable in it, so that the ion exchange between Cl^−^ and PF_6_^−^ is relatively sufficient and reversible.

### 3.3. Microscopic Morphology of Modified Paper

**Figure 8:**
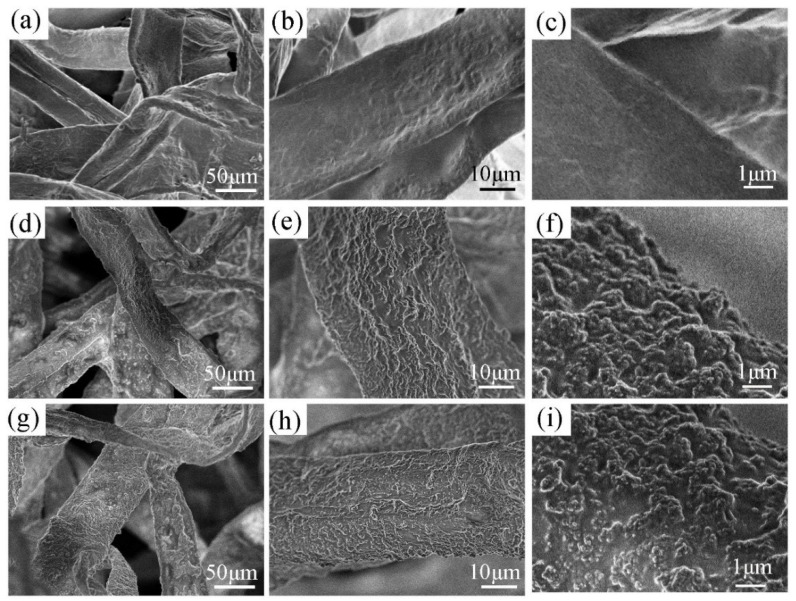
SEM images of the paper surface (**a**–**c**) Original paper; (**d**–**f**) Modified paper coated with PTSPM-PMETAC/SiO_2_-NH_2_ solution; (**g**–**i**) Modified paper that has been soaked many times.

Figure 8 shows the micro-morphology of the paper samples before and after modification. It can be seen from Figure 8a–c that the paper sample has a certain gap between the fibers before modification, which makes the paper have good air permeability, and at higher magnifications, the surface of a single paper fiber also shows a smooth state. Figure 8d–f is the graph of the modified paper fiber. As can be seen from Figure 8e,f, compared with the surface of unmodified paper fiber, the modified fiber is coated with a layer of rough polymer coating, resulting in a large number of small bumps on the fiber surface, which endows the paper with nano scale surface roughness. From Figure 8d, it could be clearly observed that the outline of the fiber has not been adhered, the surface microscopic morphology of the modified paper has not changed much, and it still maintains its good permeability and other excellent properties, which provides a very favorable condition for the construction of superhydrophobic surface [38]. Furthermore, after the modified paper was soaked in NaPF_6_ solution for 2 h and dried, the paper is also soaked in NaCl solution for 2 h and then dried, and this cycle was repeated for 5 times. At this time, the microscopic morphology of the paper surface was shown in Figure 8g–i. Compared with Figure 8d–f, the surface structure of the modified paper after anion soaking did not changed significantly, and SiO_2_ was still tightly attached to the fiber surface, retaining the original rough structure. Generally speaking, the preparation of superhydrophobic coatings needs to meet the two basic conditions of sufficient micro-rough structure and low surface energy on the surface of the substrate. In this experiment, the stable adhesion of nano-SiO_2_ on the fiber surface provided sufficient surface roughness for the paper, which also laid a solid foundation for changing the wettability of the paper surface and achieving superhydrophobic properties through anion exchange.

### 3.4. Thermogravimetric Analysis of Modified Paper

**Figure 9:**
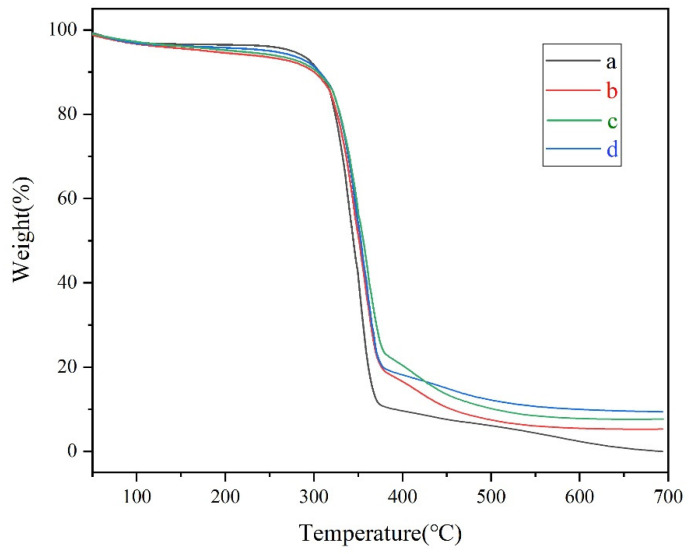
TGA cures of samples a, b, c, d: (a) Original paper; (b) PMETAC-PTSPM coated paper; (c) SiO_2_-NH_2_ coated paper; (d) Modified paper coated with PMETAC-PTSPM/SiO_2_-NH_2_. Samples were heated to 700 °C in air atmosphere at a ramp rate of 10 °C/min^−1^.

Figure 9 shows the TGA curves of paper samples a–d. Sample a is the original paper, sample b is the PMETAC-PTSPM coated paper, sample c is the SiO_2_-NH_2_ coated paper, sample d is the modified paper coated with PMETAC-PTSPM/SiO_2_-NH_2_. It can be seen from curve a that the final weight loss rate of the unmodified original paper was 100% within the tested temperature range. Due to the adhesion of the polymer on the surface of the paper, the weight loss rate of the paper became 94.7%, as shown in curve b. As shown in curve c, the modified paper coated with amino modified SiO_2_ has a weight loss rate of 91.53% and a remaining weight of 8.47%. This change in weight loss rate is mainly due to the residual SiO_2_ and the partial combustion of APTES caused. Curve d shows that after the paper was modified by PMETAC-PTSPM/SiO_2_-NH_2_ coating, the weight loss rate reached about 90.3%, and the remaining weight was 9.7%. These phenomena indicate that the coating of modified SiO_2_ on paper and the combination of PTSPM-PMETAC on the surface of modified SiO_2_ particles have affected the thermal stability of the material [39,40].

### 3.5. Stability Test

**Figure 10:**
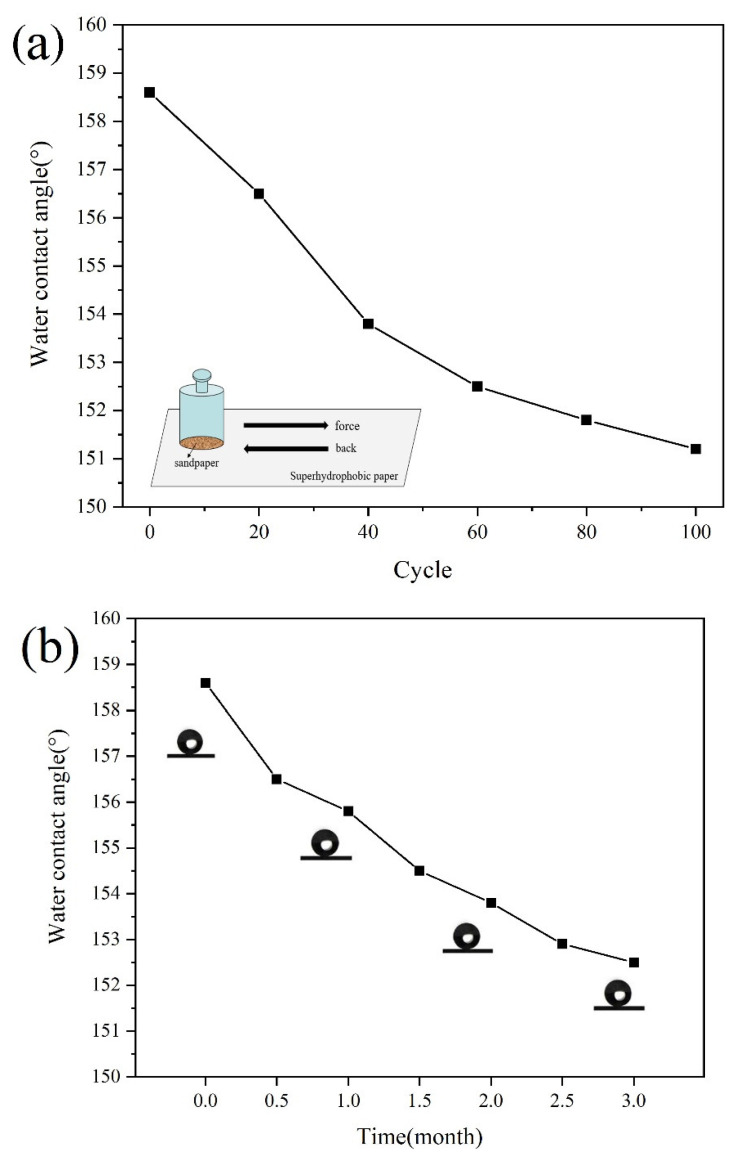
The influence of different destruction conditions on the contact angle of the paper surface (**a**) 100 rubs; (**b**) Expose outdoors for 3 months.

In the actual production and application, the destruction of external factors will lead to the decrease of the surface hydrophobicity of modified paper. Therefore, stability is an important observation index of superhydrophobic paper properties. Judging from the existing research, the currently proposed preparation methods of functional superhydrophobic surfaces tend to consider the acquisition of superhydrophobicity of the material surface and the control of wettability more, while the stability of the superhydrophobic surface was less considered. For instance, some studies have adopted ATRP reaction to graft PMETAC polyelectrolyte molecular brush on a rough silicon substrate, by changing the type of anion adsorbed by the quaternary ammonium ion in the PMETAC molecular chain, the reversible transition between superhydrophobicity and superhydrophilicity on the surface of the material can be realized. However, this method did not take into account the problem of polymer adhesion, so that the abrasion resistance and weather resistance of the prepared superhydrophobic surface were not ideal [41]. In this study, considering this problem, the stability of the superhydrophobic paper that have exchanged with NaPF_6_ ionic liquid was tested, including abrasion resistance test and weather resistance test. Figure 10a shows the changes in the contact angle of the paper surface before and after the abrasion resistance test. It can be seen from the Figure 10a that after 100 rubbing cycles on the paper surface, although the contact angle decreases slightly (the contact angle was reduced to 151.2°), it could still maintain its superhydrophobic properties, which shows that the modified paper has good abrasion resistance. Figure 10b reflects the effect of exposure time on the hydrophobic properties of paper. It can be seen that even under the condition of exposure time of 3 months, the contact angle of the modified paper only dropped from 158.6° to 152.5°, the overall fluctuation was not large, and the modified paper could still maintain the superhydrophobic properties. Therefore, the superhydrophobic paper prepared in this research has good weather resistance.

### 3.6. Transparency Test

**Figure 11:**
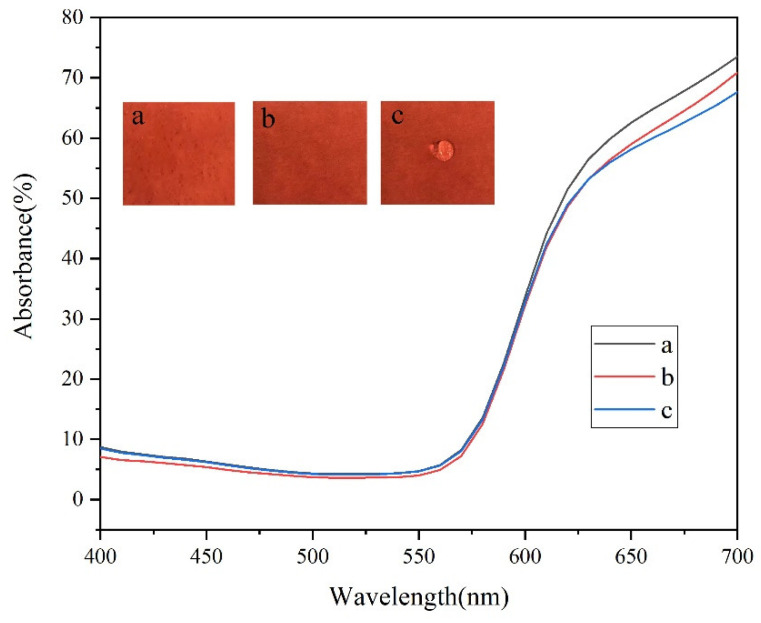
Reflectance spectra of (a) Original red paper; (b) Modified red paper; (c) Red modified paper that has been soaked many times.

The transparency of superhydrophobic coating has an important impact on its application value in packaging, construction and other fields. Although many studies have proposed some methods for the preparation of functional superhydrophobic surfaces, the transparency of superhydrophobic surface coatings was rarely analyzed [42]. This study, in order to test the transparency of the prepared superhydrophobic coating, carried out a reflective recording test. Figure 11 shows the results of the reflective recording test on three types of paper, and the transparency of the coating can be reflected by the reflective recording test data. Curve a represents the unmodified original paper, curve b represents the paper modified by PTSPM-PMETAC, and curve c represents the modified paper after NaPF_6_ soaking. In the study, while using the spectrophotometer to test the reflectance spectra of the three papers, the L, a, b values of the three papers were also tested, and the L*, a*, and b* values, quantitatively characterizing the color of the samples, were reported in Table 1. It can be seen from the Figure 11, compared with the reflectance spectrum of curve a, curves –c have similar spectral reflectance in the range of 400 nm–600 nm, while there is a slight deviation in the red spectral region of 600 nm–700 nm (color difference ∆E_b_ = 0.75, ∆E_c_ = 0.97). To the extent that the human eye can recognize, this kind of deviation is acceptable, which means that the modification and soaking of paper has little effect on its color [43,44,45]. Therefore, the prepared surface coating has good transparency.

## 4. Conclusions

In this study, PTSPM-PMETAC with anion adsorption properties was prepared by one-step method, then amino modified nano-SiO_2_ was grafted onto the polymer to improve the surface roughness and enhance the stability of superhydrophobic properties, and the superhydrophobic paper with high stability and ionic liquid-induced wettability transition was successfully prepared. The study found that this paper can achieve reversible regulation between superhydrophobicity and superhydrophilicity by adsorbing different anions in different ionic liquids through PMETAC. On this basis, the effects of ionic liquid concentration, different immersion times, and different solvents on ion exchange were discussed. The test found that water was the poor solvent for ion exchange, while the mixtures of methanol, acetone, and methanol & water were the good solvent. In addition, the preparation method of superhydrophobic paper proposed in this study not only has simple preparation process and strong universality, but also the prepared modified paper has high transparency and good stability, which has great application potential in practical productions and applications. 

## Figures and Tables

**Table 1:** The L*, a*, and b* values of **a**, **b**, **c**.

	L*	a*	b*
a	44.233	50.935	24.821
b	43.533	50.466	24.447
c	42.162	50.389	24.386

Note: **L**, **a**, **b** represent the chromaticity value of the object color: **L** represents brightness, **a** represents red-green, **b** represents yellow-blue.

## Data Availability

The data that support the findings of this study are available upon reasonable request from the authors.

## References

[1] Li J., Niu S. (2010). Life cycle comprehensive analysis assessment and limit requirements of paper packaging material. Packag. Eng..

[2] Wang N., Xiong D., Pan S., Deng Y., Shi Y., Wang K. (2016). Superhydrophobic paper with superior stability against deformations and humidity. Appl. Surf. Sci..

[3] Li H., He Y., Yang J. (2019). Fabrication of food-safe superhydrophobic cellulose paper with improved moisture and air barrier properties. Carbohydr. Polym..

[4] Youssef A.M., Samahy M.A.E., Mona H., Rehim A. (2012). Preparation of conductivepaper composites based on natural cellulosic fibers for packagingapplications. Carbohydr. Polym..

[5] Teng Y., Wang Y., Shi B. (2020). Facile preparation of economical, eco-friendly superhydrophobic surface on paper substrate with excellent mechanical durability. Prog. Org. Coat..

[6] Fu J., Yang F., Guo Z. (2019). Facile fabrication of superhydrophobic filter paper with high water adhesion. Mater. Lett..

[7] Shang Q., Chen J., Liu C. (2019). Facile fabrication of environmentally friendly bio-based superhydrophobic surfaces via UV-polymerization for self-cleaning and high efficient oil/water separation. Prog. Org. Coat..

[8] Jiang C., Liu W., Yang M., Liu C., He S., Xie Y., Wang Z. (2019). Robust multifunctional superhydrophobic fabric with UV induced reversible wettability, photocatalytic self-cleaning property, and oil-water separation via thiol-ene click chemistry. Appl. Surf. Sci..

[9] Li D., Fan J., Chen J., Jiang D. (2019). Preparation of superhydrophobic multiscale films for oil-water separation in a harsh environment. Adv. Mater. Sci. Eng..

[10] Rafik A., Elkhoshkhany N., Ahmed H., Shaker E., Aya R. (2017). High stability performance of superhydrophobic modified fluorinated graphene films on copper alloy substrates. Adv. Mater. Sci. Eng..

[11] Zhang R., Hao P., Zhang X., He F. (2018). Supercooled water droplet impact on superhydrophobic surfaces with various roughness and temperature. Int. J. Heat Mass Transf..

[12] Zhang Q., Cai S., Zhang W., Lan Y., Zhang X. (2017). Density, viscosity, conductivity, refractive index and interaction study of binary mixtures of the ionic liquid 1–ethyl–3–methylimidazolium acetate with methyldiethanolamine. J. Mol. Liq..

[13] Lahann J., Mitragotri S., Tran T.N., Kaido H., Langer R. (2003). A reversibly switching surface. Science.

[14] Geissler A., Loyal F., Biesalski M., Zhang K. (2014). Thermo-responsive superhydrophobic paper using nanostructured cellulose stearoyl ester. Cellulose.

[15] Wang L., Lin Y., Peng B., Su Z. (2009). Tunable wettability by counterion exchange at the surface of electrostatic self-assembled multilayers. Chem. Commun..

[16] Jiang W., Wang G., He Y., Wang X., An Y., Song Y., Jiang L. (2005). Photo-switched wettability on an electrostatic self-assembly azobenzene monolayer. Adv. Commun..

[17] Welton T. (2018). Ionic liquids: A brief history. Biophys. Rev..

[18] Singh S., Savoy A. (2020). Ionic liquids synthesis and applications: An overview. J. Mol. Liq..

[19] Ahrenberg M., Beck M., Neise C., Keßler O., Kragl U., Verevkincd S., Schick C. (2016). Vapor pressure of ionic liquids at low temperatures from AC-chip-calorimetry. Phys. Chem. Chem. Phys..

[20] Wu H., Zhang B., Liu S., Chen C. (2020). Flammability estimation of 1-hexyl-3-methylimidazolium bis(trifluoromethylsulfonyl)imide. J. Loss Prev. Process Ind..

[21] Clarke C., Le L., Hallett J., Licence P. (2020). Thermally-Stable Imidazolium Dicationic Ionic Liquids with Pyridine Functional Groups. ACS Sustain. Chem. Eng..

[22] Mero A., Mezzetta A., Nowicki J., Łuczak J., Guazzelli L. (2021). Betaine and L-carnitine ester bromides: Synthesis and comparative study of their thermal behavior and surface activity. J. Mol. Liq..

[23] Mezzetta A., Becherini S., Pretti C., Monni G., Casu V., Chiappe C., Guazzelli L. (2019). Insights into the levulinate-based ionic liquid class: Synthesis, cellulose dissolution evaluation and ecotoxicity assessment. New J. Chem..

[24] Karmakar A., Mukundan R., Yang P., Batista E. (2019). Solubility model of metal complex in ionic liquids from first principle calculations. RSC Adv..

[25] Keaveney S., Haines R., Harper J. (2017). Ionic liquid solvents: The importance of microscopic interactions in predicting organic reaction outcomes. Pure Appl. Chem..

[26] Calmanti R., Selva M., Perosa A. (2020). Tungstate ionic liquids as catalysts for CO_2_ fixation into epoxides. Mol. Catal..

[27] Claus J., Sommer F., Kragl U. (2018). Ionic liquids in biotechnology and beyond. Solid State Ion..

[28] Martins V., Torresi R. (2018). Ionic liquids in electrochemical energy storage. Curr. Opin. Electrochem..

[29] Trujillo-Rodríguez M., Nan H., Varona M., Emaus N.M., Anderson J.L. (2019). Advances of Ionic Liquids in Analytical Chemistry. Anal. Chem..

[30] Pedro S., Freire C., Silvestre A., Freire M. (2020). The Role of Ionic Liquids in the Pharmaceutical Field: An Overview of Relevant Applications. Int. J. Mol. Sci..

[31] Zhang Q., Jin B., Wang B., Fu Y., Zhan X., Chen F. (2017). Fabrication of a Highly Stable Superhydrophobic surface with dual-scale structure and its antifrosting properties. Ind. Eng. Chem. Res..

[32] Fu Y., Jiang J., Zhang Q., Zhan X., Chen F. (2017). Robust liquid-repellent coatings based on polymer nanoparticles with excellent self-cleaning and antibacterial performances. J. Mater. Chem. A.

[33] Jiang S., Zhou S. (2021). A method for preparing the pH-responsive superhydrophobic paper with high stability. Mater. Res. Express..

[34] Azzaroni O., Brown A.A., Huck W.T.S. (2007). Tunable wettability by clicking counterions into polyelectrolyte brushes. Adv. Mater..

[35] Yin Y., Guo N., Wang C., Rao Q. (2014). Alterable Superhydrophobic–Superhydrophilic Wettability of Fabric Substrates Decorated with Ion–TiO2 Coating via Ultraviolet Radiation. Ind. Eng. Chem. Res..

[36] Hojo M., Ueda T., Ueno E., Hamasaki T., Nakano T. (2010). Salt effects on the rates and mechanisms of solvolysis reaction of organic halides and water structure distortion in N, N-dimethylformamide-and N, N-dimethylacetamide-water mixed solvents. Bull. Chem. Soc. Jpn..

[37] Okada T., Harada M. (2004). Hydration of halide anions in ion—exchange resin and their dissociation from cationic groups. Anal. Chem..

[38] Du B., Chen F., Luo R., Li H., Zhou S., Liu S., Hu J. (2019). Superhydrophobic surfaces with pH-induced switchable wettability for oil−water separation. ACS Omega.

[39] Jiang S., Zhou S., Du B., Luo R. (2021). Preparation of superhydrophobic paper with double-size silica particles modified by amino and epoxy groups. AIP Adv..

[40] Xue C., Jia S., Zhang J., Tian L., Chen H., Wang M. (2008). Preparation of superhydrophobic surfaces on cotton textiles. Natl. Inst. Mater. Sci..

[41] Lim H., Lee S., Lee D., Lee D., Lee S., Cho K. (2008). Superhydrophobic to Superhydrophilic Wetting Transition with Programmable Ion-Pairing Interaction. Adv. Mater..

[42] Ge H., Wang G., He Y., Wang X., Song Y., Jiang L., Zhu D. (2006). Photo switched Wettability on Inverse Opal Modified by a Self-Assembled Azobenzene Monolayer. Chem. Phys. Chem..

[43] Jiang S., Zhou S., Du B., Luo R. (2021). Preparation of the temperature-responsive superhydrophobic paper with high stability. ACS Omega.

[44] Yazdanshenas M.E., Shateri M. (2013). One-Step synthesis of superhydrophobic coating on cotton fabric by ultrasound irradiation. Ind. Eng. Chem. Res..

[45] Jiang S., Zhou S., Du B., Luo R. (2020). A study on the stability of superhydrophobic paper reinforced by amino-assisted modified PHFMAPTSPM polymer. Mater. Res. Express.

